# Perspectives on Telehealth for older adults during the COVID-19 pandemic using the quadruple aim: interviews with 48 physicians

**DOI:** 10.1186/s12877-022-02860-8

**Published:** 2022-03-08

**Authors:** Elizabeth M. Goldberg, Michelle P. Lin, Laura G. Burke, Frances N. Jiménez, Natalie M. Davoodi, Roland C. Merchant

**Affiliations:** 1 Department of Emergency Medicine, The Warren Alpert Medical School of Brown University, 55 Claverick Street, Second floor, Providence, RI 02903 USA; 2 Department of Health Services, Policy and Practice, Brown University School of Public Health, Providence, RI USA; 3 Department of Emergency Medicine, Icahn School of Medicine at Mount Sinai, New York, NY USA; 4 Department of Population Health Science and Policy, Icahn School of Medicine at Mount Sinai, New York, NY USA; 5 Department of Emergency Medicine, Beth Israel Deaconess Medical Center, Boston, MA USA; 6 Brown University School of Public Health, Providence, RI USA

**Keywords:** Telehealth, Qualitative research, Geriatricians, Primary care, COVID-19

## Abstract

**Background:**

Telehealth delivery expanded quickly during the COVID-19 pandemic after the reduction of payment and regulatory barriers, but older adults are the least likely to benefit from this expansion. Little is known about physician experiences initiating telehealth and factors that fostered or discouraged adoption during the COVID-19 pandemic with older adult patients. Therefore, our objective was to understand experiences of frontline physicians caring for older adults via telehealth during the COVID-19 pandemic.

**Methods:**

We conducted semi-structured interviews from September 2020 to November 2020 with 48 physicians. We recruited a diverse sample of geriatricians (*n* = 18), primary care (*n* = 15), and emergency (n = 15) physicians from all United Stated (US) regions, rural-urban settings, and academic-community practices who cared for older adult patients during the pandemic using purposive sampling methods. We completed framework analysis of the transcribed interviews to identify emerging themes and used the Quadruple Aim to organize themes.

**Results:**

Frontline physicians described telehealth as a more flexible, value-based, and patient-centered mode of health care delivery. Benefits of using telehealth to treat older adults included reducing deferred care and increasing timely care, improving efficiency for physicians, enhancing communication with caregivers and patients, reducing patient travel burdens, and facilitating health outreach and education. Challenges included unequal access for rural, older, or cognitively impaired patients. Physicians noted that payment parity with in-person visits, between video and telephone visits, and relaxation of restrictive regulations would enhance their ability to continue to offer telehealth.

**Conclusions:**

Frontline physicians who treated older adults during the COVID-19 pandemic were largely in favor of continuing telehealth use beyond the pandemic; however, they noted that sustainability would depend on enacting policies that address access inequities and reimbursement concerns. Our data provide policy insights that if placed into action could facilitate the long-term success of telehealth and encourage a more flexible healthcare delivery system in the US.

**Supplementary Information:**

The online version contains supplementary material available at 10.1186/s12877-022-02860-8.

## Background

In the United States (US), the coronavirus disease (COVID-19) pandemic promulgated a 154% increase in telehealth visits in March 2020 compared to March 2019 [1]. This rapid uptake of telehealth was accelerated by deregulation of payment and regulatory policy. The Centers for Medicare and Medicaid Services (CMS) and many private health insurance payers allowed payment parity between in-person and virtual visits [2]. Medicare expanded coverage for telehealth by (1) waiving the audio-video requirement for certain telehealth services, (2) introducing payment for remote physiologic monitoring [3], and (3) allowing hospitals to bill for services furnished remotely by hospital-based clinicians. Health Insurance Portability and Accountability Act (HIPAA) violation penalties were suspended if telehealth was provided in good faith, permitting use of Facetime and other non-HIPAA-compliant platforms [4]. Relaxation of state licensing requirements allowed for out-of-state physicians to practice in more jurisdictions [5].

Older adults (aged 65 years and older) are the most vulnerable to severe complications and death due to COVID-19 [6], however they are also less likely to benefit from expanded telehealth access and use [7–10]. While the older adult population spans an age group of many decades with varied abilities and health concerns, older adults tend to face greater barriers to technology and internet use compared to younger adults. Notable barriers include internet and device access, design challenges, privacy and trust concerns, and cost [11]. Yet, technology use among older adults is growing in the US [12], and 27% of Medicare beneficiaries accessed telehealth during the COVID-19 pandemic [13].

Expanded telehealth use has the potential to permanently transform healthcare delivery systems and access to care for older adult populations facing challenges to in-person care [14], as well as improving chronic condition management [15], and reducing healthcare costs [16]. However, little is known about physician experiences initiating telehealth and system-level and contextual factors that fostered or discouraged adoption during the COVID-19 pandemic and implications of use beyond the pandemic.

The Quadruple Aim, a conceptual framework that optimizes health system performance, seeks to guide development of high value care for patients while encouraging a transition to population health through four domains: patient care experience, population health, healthcare costs, and workforce engagement and safety [17, 18]. Examining physicians’ experiences using telehealth with older adults within the domains of the Quadruple Aim could be useful to improve health care delivery for older adults beyond the pandemic. Specifically, this study sought to understand the experiences of geriatricians, primary care physicians (PCPs), and emergency medicine physicians – who provide most acute and chronic care for US older adults [19] – in relation to telehealth use with older adults. Therefore, we interviewed these frontline physicians on the impact of telehealth policy changes during the COVID-19 pandemic on telehealth access and the older patient experience using telehealth from the physician perspective according to the Quadruple Aim.

## Methods

### Summary

We conducted semi-structured virtual interviews with geriatricians, primary care, and emergency physicians from diverse settings. Telehealth was defined broadly as remote communication with patients, including telephone calls, audiovisual visits, home monitoring with wearables (i.e., blood pressure monitors), web portals, or app-based communication. The principal investigator’s hospital’s Institutional Review Board (IRB) approved the study. We follow the Consolidated Criteria for Reporting Qualitative Research (COREQ) [20] in reporting our findings.

### Sample

Study eligible physicians were licensed to practice in the US in geriatrics, primary care (internal medicine or family practice), or emergency medicine (EM). Because we sought to understand physician experiences caring for older patients, given their risk for COVID-19 isolation and morbidity, participants must have cared for patients 65 years and older in-person or via telehealth during the pandemic.

### Recruitment

We stratified recruitment by specialty and practice type (academic and community) to overcome limitations of prior telehealth research that primarily included physicians from large academic health systems [21]. Using purposive sampling, we solicited physicians on two social media platforms (Twitter, Facebook), which 75–90% of physicians use [22, 23], and two specialty society listservs (Academy for Geriatric Emergency Medicine (158 members) and the American Geriatric Society Member Forum (7600 members)). We posted our flyer once on each social media platform and listserv using the first author’s account.

### Interview content

We developed an interview guide which contained a description of study objectives and open-ended questions with probes for follow-up questions (Additional file 1: Appendix 1). Questions asked about physicians’ general experiences with adopting and using telehealth during the pandemic, their preferred modality (e.g., phone call, web portals, etc.), and their motivations/barriers to using telehealth. Additional questions focused on older patient telehealth access, acceptability, and experience, acknowledging that older adults faced unique challenges during the pandemic and with using technology for health purposes. Questions were pilot tested among the research staff before use.

### Interview procedure

The first and fourth authors conducted video interviews via Zoom (Zoom Video Communications Inc., 2016). The first author is a female physician with formal qualitative research training and was known to some participating physicians. The fourth author is a female graduate student with 3 years of professional experience in qualitative research techniques. After obtaining verbal consent, interviews were audio-recorded, transcribed, and de-identified. The interviewer completed a written debriefing after each interview to document findings such as new or emerging themes and additional questions that should be added. Participants received a gift card after completing the interviews. No repeat interviews were conducted. Participant feedback was not sought. No one refused or dropped out of interviews.

### Analysis

We used *framework analysis* that involved summarizing content within categories into charts after transcription; this approach allows for the systematic analysis of participant perspectives and the incorporation new codes to a priori codes developed based on the study protocol [24, 25]. We performed the following steps according to this analytic framework: (1) We *familiarized ourselves* with the data through reviewing and re-rereading the transcripts. (2) We developed a set of a priori *codes* based on our interview questions and study protocol. (3) We reviewed the coding of the initial three transcripts line-by-line with the entire research team until reaching agreement on the codes to apply to all subsequent transcripts. (4) The working analytical framework was applied by indexing the remaining transcript using the existing codes. All transcripts were double coded in rotating pairs by a team of 10 researchers. We used NVivo (Version 16) to organize the coded data [26]. (5) We *charted* data by summarizing it and organizing it within categories in a spreadsheet. All summaries were reviewed by an interviewer to ensure consistency across team members in charting technique and maintenance of the original meaning of the interview. (6) We iteratively *searched for common themes and subthemes across participants*. (7) We reviewed *themes* in relation to the entire dataset and selected representative quotes from the interviews to illustrate the themes. The Quadruple Aim was used to further group themes into the following four domains: care experience, older individuals’ health, cost, and workforce engagement. Key steps and coding decisions were recorded in an ongoing *audit trail* [27].

## Results

We interviewed 18 geriatricians, 15 primary care, and 15 emergency physicians between September and November 2020. Median participant age was 37.5 years-old with a median of 7 years in clinical practice. Table 1 describes demographic characteristics of study participants. Interviews lasted a mean of 30 min. Overall, participants expressed mixed perspectives on the adoption of telehealth for the care of older adults. Six major themes emerged, all which relate to domains of the Quadruple Aim: the patient experience of care, health of populations, cost, and workforce engagement [17, 18].

**Table 1:** Interviewee demographic characteristics and telehealth use prior to and during COVID-19 pandemic, for total sample and by specialty

	Total (*n* = 48)	Geriatrics^a^ (*n* = 18)	Primary Care^c^ (*n* = 15)	Emergency Medicine^b^ (*n* = 15)
**Age (years)**				
25–44	36 (75.0)	11 (61.1)	13 (86.7)	12 (80.0)
45–64	7 (14.6)	3 (16.7)	1 (6.7)	3 (20.0)
65 and over	5 (10.4)	4 (22.2)	1 (6.7)	0 (0.0)
**Sex**				
Male	21 (43.8)	10 (55.6)	3 (20.0)	8 (53.3)
Female	27 (56.2)	8 (44.4)	12 (80.0)	7 (46.7)
**Years in Medical Practice**				
0–10	33 (68.8)	10 (55.6)	11 (73.3)	11 (73.3)
11–21	9 (18.8)	2 (11.1)	3 (20.0)	4 (26.7)
22–32	2 (4.2)	2 (11.1)	1 (6.7)	0 (0.0)
33 years or more	4 (8.3)	4 (22.2)	0 (0.0)	0 (0.0)
**Region**				
Northeast	19 (39.6)	6 (33.3)	4 (26.7)	9 (60.0)
Midwest	10 (20.8)	3 (16.7)	3 (20.0)	4 (26.7)
South	9 (18.8)	5 (27.8)	3 (20.0)	1 (6.7)
West	10 (20.8)	4 (22.2)	5 (33.3)	1 (6.7)
**Practice Setting**				
Metro	26 (54.2)	12 (66.7)	7 (46.7)	7 (46.7)
Suburban	18 (37.5)	4 (22.2)	8 (53.3)	6 (40.0)
Rural	4 (8.3)	2 (11.1)	0 (0.0)	2 (13.3)
**Practice Type**				
Academic	24 (50.0)	9 (50.0)	5 (33.3)	10 (66.7)
Community	24 (50.0)	9 (50.0)	10 (66.7)	5 (33.3)
**Telehealth use prior to COVID-19 pandemic**				
Video-visit only	8 (16.7)	2 (11.1)	3 (16.7)	3 (16.7)
Non-video visit only	14 (29.2)	5 (27.8)	6 (40.0)	3 (16.7)
Video and non-video visits	6 (12.5)	2 (11.1)	1 (6.7)	3 (16.7)
No telehealth	20 (41.7)	9 (50.0)	5 (33.3)	6 (40.0)
**Estimated no. of telehealth visits completed during pandemic**^d^**, median (IQR)**	224 (64–640)	250 (64–640)	500 (200–960)	100 (35–400)

*Abbreviations*: *IQR* Interquartile range

^a^Some geriatricians reported a secondary specialty: Hospice and Palliative Medicine (n = 1); Sleep Medicine (n = 1). ^b^ Some emergency medicine physicians reported a secondary specialty: Clinical Informatics (n = 1); Internal Medicine (*n* = 1). ^c^ Primary care physicians were boarded in Internal Medicine (*n* = 12) or Family Medicine (*n* = 3). Some primary care physicians reported a secondary specialty: Clinical Information (n = 1); Geriatrics (*n* = 2); Pediatrics (n = 1); Sports Medicine (n = 1). ^d^Estimated pandemic period was 32 weeks between March 13 and October 16, 2020

### Theme 1: Telehealth could transform care delivery, but equitable access must be addressed (quadruple aim domain: care experience)

Many physicians described that telehealth altered the way they delivered care by making it more efficient, more practical for patients, and allowing more opportunities to connect with patients (Additional file 1: Appendix 2). App-based text messaging platforms facilitated communication with multiple patients concurrently and allowed for more in-depth conversations in emergency department (ED) settings. Many physicians described that telehealth enhanced communication with patients and caregivers and reduced travel burden for older adult patients with mobility issues. Some emergency physicians reported that telehealth helped patients avoid long waits as hospitals were responding to surges in cases. Office-based physicians pointed out that telehealth improved their ability to offer repeat visits for patients with undifferentiated conditions.

Beyond improving the patient-facing access barriers mentioned above, physicians also reported telehealth alleviated pandemic-related office closures and staff shortages. Geriatricians noted that telehealth could relieve physician staffing concerns at facilities, thereby improving access to care for older adults and could make telehealth “a major part of nursing home visits going forward” (Interview 9, geriatrician, Northeast, community). Physicians reported that the benefits in access to care posed by telehealth “trumps the potential pitfalls” (Interview 48, PCP, South, community). Many physicians hoped to continue providing telehealth as a supplement to in-person care in the future and reported that their patients had grown accustomed to having telehealth as an option.

However, some participants reported barriers to care due to telehealth. Video telehealth requires web-enabled devices that can be unaffordable to older patients. Several physicians also noted that helping older patients use telehealth for the first time was time consuming, costly, and frustrating. Some reported having to retrain staff, hire new staff, or rely on student volunteers to provide a pre-visit technology orientation to patients. While many feared that older adults may have difficulty using digital health technology, several physicians felt that digital literacy was not a significant barrier for caring for older patients and that as younger generations age, telehealth will be “here to stay” (Interview 6, geriatrician, West, community).

### Theme 2: regulatory and policy changes are necessary to improve the older patient experience (quadruple aim domain: care experience)

Participants stated that regulatory changes are needed to improve older patient access to telehealth and ensure sustainability (Additional file 1: Appendix 2). One physician shared that policymakers should consider “how many people lack home internet [and] connected devices,” so when “equitable policies” for those “marginalized in society” are designed, “audio-only diagnoses” are appreciated as the primary way to reach many older Americans (Interview 34, geriatrician, South, community).

Physicians reported a need for changes in patient privacy laws. Physicians stated that patients valued privacy, but HIPAA regulations often thwarted technological innovation. One PCP stated patients “don’t give a flippa about HIPAA” (Interview 20, PCP, West, community); patients simply want to connect in the easiest, most seamless fashion to obtain necessary medical care. Some acknowledged that their patients had security concerns about “Zoom bombing,” or disruptive intrusions by hackers, but no participants shared examples of actual privacy violations that occurred. Many acknowledged FaceTime was preferable to HIPAA-compliant videoconferencing platforms due to increased patient access and reduced complexity.

Other recommendations for improvement included enhancing medicolegal protections. Several emergency physicians noted that while telehealth improved access, it also created diagnostic uncertainty with liability implications for missing symptoms due to the virtual format. In general, physicians also requested that federal and state officials remove restrictive licensure and credentialing because they were neither aligned with how patients prefer to seek care nor addressed the realities of physician shortages in many US states. Emergency physicians stated they would like to work occasionally, or “moonlight”, in rural or out-of-state hospitals, but felt restricted due to the need for expensive, time-intensive credentialing procedures. This concern was highlighted by participants situated near state borders, where patients from other states could drive across the border and be seen in-person; however, they were unable to provide care to these same patients virtually due to licensing laws. Multiple participants observed that during the pandemic some states reduced these barriers. One geriatrician stated that under emergency licensure their home health agency expanded their patient panel to include out-of-state patients, which was not possible previously. One PCP commented that certifying home health through virtual visits is more patient-centric: “It’s less stress for families having to haul either [patients with dementia] or really immobile patients into the office. But still be able to get the care that they need” (Interview 40, PCP, West, community).

### Theme 3: Telehealth could improve older adults’ health by enhancing access to low-barrier care (quadruple aim domain: older individuals’ health)

Physicians reported that telehealth enhanced access to low-barrier care by reducing deferred care and increasing timely care, reducing spread of communicable diseases, strengthening communication with caregivers, and facilitating health outreach and education, thus improving older patient health (Additional file 1: Appendix 3). Some physicians described that without telehealth, patients would have completely forgone visits during the pandemic, with potentially devastating consequences on individuals’ health. In some instances, telehealth enabled physicians to prevent clinical decomposition and recommend patients seek necessary in-person medical care when patients had been otherwise unconcerned.

By increasing access to low-barrier care, physicians saw telehealth as a tool to protect patients and healthcare workers by mitigating the spread of communicable diseases, particularly when personal protective equipment was in short supply. Geriatricians noted that telehealth facilitated communication with patients in assisted living and nursing homes, which were under strict quarantine rules. Some physicians also reported that adopting telehealth during the COVID-19 pandemic would make them more willing to use telehealth in the future to prevent spread of other communicable diseases, such as influenza.

Further, physicians were able to employ telehealth to advance public health efforts and provide COVID-19-related education to patients. Emergency medicine physicians reported that asynchronous, chat-based visits often centered around explaining public health guidance, combatting misinformation, and directing patients to testing centers when there was reduced accessibility to primary care and concerns about the safety of seeking care in hospitals.

### Theme 4: Telehealth has potential to result in cost savings so long as unnecessary referrals and tests are avoided (quadruple aim domain: cost)

Physicians of all specialties cited opportunities for telehealth to provide cost savings and reduce system strain, including by reducing avoidable specialist consultations, hospital or urgent care use, and testing (Additional file 1: Appendix 4). However, some physicians noted that the lack of a hands-on examination might lead them to order unnecessary referrals and additional testing, which could increase healthcare costs. Physicians who reported increasing specialist referrals often cited doing so over concerns of misdiagnosis since physical examinations were not possible via telehealth.

### Theme 5: cost-related factors were cited as the driving force in telehealth adoption (quadruple aim domain: cost)

Some physicians believed that funding, not acceptability of telehealth, was the primary factor in telehealth adoption and sustainability, because patient and provider satisfaction with telehealth was high (Additional file 1: Appendix 4). Many hospital-based physicians mentioned that their employers were concerned telemedicine would disrupt current payment models by reducing facility fees and reimbursement for care, sometimes putting employers at odds with physician and patient telehealth preferences. Some physicians stated that they were pressured by administrators and organizational leadership not to offer telephone visits due to lower reimbursement of telephone compared to video or in-person visits. There were often repercussions for physicians who offered telephone visits, which many still did because the alternative was to deny treatment or ask patients to schedule in-person visits when they may have been uncomfortable doing so due to the risk of disease transmission.

Several physicians practicing in fee-for-service models stated that telephone calls should be reimbursed at similar rates to video visits, or they would need to abandon telehealth entirely. One physician noted that adequate reimbursement for non-video telehealth modalities, like audio-only and text-based platforms, “almost exclusively drives whether [they’re] going to exist or not and without getting reimbursed, [they’ll] just evaporate again” (Interview 35, EM, Northeast, community). Providing non-video telehealth appointments would address telehealth access concerns, as many patients could not participate in video visits due to lacking digital literacy, internet connectivity, or devices. For patients with mobility or transportation issues that make in-person visits undesirable, physicians noted that being patient-centered would mean allowing the patient to choose what mode of telehealth to use rather than restricting them to options that are reimbursed at a higher rate, such as video or in-person visits. For these reasons, some physicians practicing in a fee-for-service setting observed that widespread adoption of telehealth has the potential to transform healthcare financing and delivery, if sustained beyond the pandemic. One PCP noted that telehealth could “break the fee-for-service world” if progress made during the COVID-19 pandemic continued (Interview 2, PCP, West, community).

In general, physicians in practices employing value-based models faced fewer barriers initiating telehealth and using patient-preferred telehealth modalities (e.g., telephone calls), which allowed for earlier or more seamless adoption of telehealth during the pandemic. Physicians also saw opportunities to provide innovative treatment models based in value and not volume, such as partnering with paramedic services to avoid unnecessary trips to the ED. Many physicians acknowledged that fee-for-service reimbursement models underappreciate the value of patient counseling, observation of clinical conditions, and time needed to address complex psychosocial patient concerns, at the expense of high-quality patient care. If telehealth visits were reimbursed adequately, physicians saw telehealth as a clinical strategy to ensure dedicated time to patient counseling and care continuity.

### Theme 6: Telehealth was beneficial for workforce engagement (quadruple aim domain: workforce engagement)

Physicians expressed that reimbursement for telehealth should continue beyond the pandemic as current relaxed policies “assigned value” for previously uncompensated work (Interview 40, PCP, West, community). Multiple office-based physicians reported satisfaction with the temporary payment changes, with one physician stating, “I feel like I was already doing all this work, but not getting reimbursed … I’m so much happier [now]” (Interview 44, PCP, Midwest, community). Because of this, many physicians reported a desire to continue providing telehealth visits as a supplement to their in-person practice beyond the pandemic (Additional file 1: Appendix 5).

Additionally, some physicians reported higher satisfaction with telehealth than their pre-pandemic schedule due to improved work-life balance. In particular, physicians with families stated that their quality of life had improved with the introduction of telehealth because they were able to be more involved in their family life and have time to exercise and prepare meals. However, some older physicians stated they missed in-person contact with patients and colleagues and this had a negative impact on morale.

## Discussion

In this investigation exploring physician perspectives on the use of telehealth during the COVID-19 pandemic, several key themes relating to the care experience, older individuals’ health, cost, and workplace engagement were revealed. Findings suggest that the flexible telehealth payment policies developed out of necessity during the public health crisis have highlighted existing shortcomings in the US healthcare delivery system yet revealed promising solutions [28]. Clinicians, researchers and policymakers have long noted the degree to which the prevailing fee-for-service delivery system fails to provide the most effective, efficient, or patient-centered care [29, 30]. Lower-intensity services that have historically been poorly reimbursed or not reimbursed at all, such as family meetings and telephone visits, could be the most effective and convenient for the patient [31, 32]. Yet, clinicians who have engaged in these practices under the traditional fee-for-service model are not compensated for their time and may even be professionally penalized. Studies have linked physician burnout to the growing number of uncompensated tasks needed to meet patients’ needs while also maintaining a certain volume of billable encounters [33, 34]. Our findings suggest that in the absence of sustainable payment parity, telehealth may negatively impact physician retention and may exacerbate burnout in the short and long term.

Indeed, several physicians in this study experienced pressure from their respective organization’s leadership to limit telephone visits, which reimbursed less compared to in-person or video visits, even to patients who could not easily access care in other ways. These findings suggest that flexible payment policy and attention to key details (e.g., the mode of telehealth delivery) can help align organizational financial incentives with patients’ needs and providers’ professional ethics.

Study participants across specialties and care settings detailed the unique ways in which the flexibility of telehealth during the COVID-19 pandemic improved patient care and their own professional satisfaction. Specifically, long-term payment parity for telehealth services relative to in-person care was repeatedly cited by participants as a requisite for long-term adoption. Of note, participants already engaged in alternative payment models, including accountable care organizations, Veterans’ Affairs, and Kaiser Permanente, experienced the most seamless adoption of telehealth. Future alternative payment designs could consider investments into telehealth infrastructure in population health payments, with higher payments to providers caring for patients with disproportionately lower access to telehealth services. Taken together, these findings suggest that the marked growth in telehealth adoption during the pandemic has provided a much-needed catalyst to address pre-existing challenges and accelerate the trend toward alternative payment models. Further research is needed to investigate the impact of low-cost services such as telehealth on overall healthcare utilization.

Yet, while the overall experience of study participants with telehealth during the COVID-19 pandemic was largely positive, several challenges were noted which highlight other longstanding barriers to care delivery in the US. The concerns about telehealth access among marginalized patients and the challenges for the clinicians who disproportionately treat them is reminiscent of past concerns that even well-intended programs have the potential to widen disparities in healthcare access and outcomes [7]. Particular attention should be paid to the potential for telehealth to widen existing disparities among patients of low digital literacy, who are often also likely to be low-income minorities [35], or older adults [8, 36–38] and those living in rural areas with limited broadband infrastructure [39, 40].

Several regulatory and policy changes are necessary to ensure the sustainability of telehealth. The temporary relaxation of state licensing requirements alleviated staffing shortages during the COVID-19 pandemic, both due to underlying variation in clinician density and due to differing impact of COVID-19 among US regions over time. The restoration of in-state licensing and credentialing may create unnecessary hurdles for providers seeking to provide telehealth care in the settings with the greatest shortages, such as rural communities [41], where the challenges in traditional healthcare access [42] and outcomes [43] have been well-documented. The temporary waiver of HIPAA to allow physicians to use their personal cellular phones (e.g., FaceTime) reduced technological barriers to telehealth adoption. The Privacy Rules within HIPAA need revision to support—and not hinder—patient privacy and access to care; HIPAA was enacted in 1996 [44], well before the widespread adoption of modern technologies that support telehealth, such as video communication platforms.

While we found positive impacts of telehealth on patient experience overall, additional research needs to be done to examine patient-centered outcomes under telehealth. While telehealth may increase efficiency in some settings, it may also lead to unintended adverse consequences such as increased specialist referrals. Many participants expressed concerns that telehealth could increase costs because they feared litigation related to misdiagnosis without a hands-on physical exam, suggesting need for further training on appropriate uses for telehealth with older patients. Future research should explore appropriate telehealth use and build the evidence-base on adapting in-person examinations for virtual settings.

### Limitations

Our results reflect the experience of participant physicians from the first 6 months of the pandemic and might not be representative of its later phases. Physicians were from select specialties, and may not reflect experiences of physicians from other specialties or non-physicians. Patients and non-physician staff are important stakeholders in clinical practice transformation, and future research should explore their perspectives. Younger physicians with prior telehealth use may have been more likely to respond to our flyer. As such, more favorable views of telehealth may have been captured and results may not be generalizable to older physicians or those with less technology use. However, we found themes were similar among younger and older physicians in our sample. Although our study included only four (8%) rural physicians, in 2000 only 9% of US physicians practiced in rural areas [45]. We did not interview rural PCPs, and opinions of rural emergency medicine physicians and geriatricians interviewed may vary from rural PCPs.

## Conclusion

In summary, participating physicians largely described telehealth as having the capacity to reform healthcare delivery to be more flexible, value-based, and patient-centered. They identified several benefits and still existing challenges of telehealth relating to the Quadruple Aim and cautioned that the continued success of telehealth will depend on key policy and regulatory decisions. These physicians also expressed optimism and specific ideas of how telehealth adoption during the COVID-19 pandemic could catalyze healthcare delivery transformation in the years to come. Our data provide policy insights that, if placed into action, could facilitate the long-term success of telehealth and encourage a more flexible healthcare delivery system in the US.

## Supplementary Information

**Additional file 1: Appendix 1.** Interview Protocol. **Appendix 2: Table 2.** Themes and Representative Quotations from Interviews in the Study of Geriatricians, Primary and Emergency Care Physicians on Telehealth, 2020 (Domain: Care Experience). **Appendix 3: Table 3.** Themes and Representative Quotations from Interviews in the Study of Geriatricians, Primary and Emergency Care Physicians on Telehealth, 2020 (Domain: Older Individuals’ Health). **Appendix 4: Table 4.** Themes and Representative Quotations from Interviews in the Study of Geriatricians, Primary and Emergency Care Physicians on Telehealth, 2020 (Domain: Cost). **Appendix 5: Table 5.** Themes and Representative Quotations from Interviews in the Study of Geriatricians, Primary and Emergency Care Physicians on Telehealth, 2020 (Domain: Workforce Engagement).

## Data Availability

Human subjects protections preclude us from granting public access to participant-level data. Access to the interview guide and select interview quotations are available in the supplementary material. Full interview transcripts cannot be released due to their identifiable nature.

## Abbreviations

US: United States
COVID-19: Coronavirus disease
CMS: Centers for Medicare and Medicaid Services
HIPAA: Health Insurance Portability and Accountability Act
EM: Emergency medicine
PCPs: Primary care physicians
IRB: Institutional Review Board
COREQ: Consolidated Criteria for Reporting Qualitative Research
ED settings: Emergency department
IQR: Interquartile range
APP: Advanced practice provider
DME: Durable medical equipment
EMS: Emergency medical services
ER: Emergency room
RVU: Relative value unit
