# Supplementary material for: Perspectives on Telehealth for older adults during the COVID-19 pandemic using the quadruple aim: interviews with 48 physicians
Source: BMC Geriatr. 2022 Mar 8;22:188. doi: 10.1186/s12877-022-02860-8 (PMC8903127; doi:10.1186/s12877-022-02860-8)
Supplement: Supplementary file 1 — Additional file 1: Appendix 1. Interview Protocol. Appendix 2: Table 2. Themes and Representative Quotations from Interviews in the Study of Geriatricians, Primary and Emergency Care Physicians on Telehealth, 2020 (Domain: Care Experience). Appendix 3: Table 3. Themes and Representative Quotations from Interviews in the Study of Geriatricians, Primary and Emergency Care Physicians on Telehealth, 2020 (Domain: Older Individuals’ Health). Appendix 4: Table 4. Themes and Representative Quotations from Interviews in the Study of Geriatricians, Primary and Emergency Care Physicians on Telehealth, 2020 (Domain: Cost). Appendix 5: Table 5. Themes and Representative Quotations from Interviews in the Study of Geriatricians, Primary and Emergency Care Physicians on Telehealth, 2020 (Domain: Workforce Engagement). [file 12877_2022_2860_MOESM1_ESM.docx]

**Appendix 1. Interview Protocol**

**Aim 1 Physician Questionnaire**

This interview agenda is a guide for the interviewers, but not a rigid script. We have added intent statements explaining the goals of a particular line of questioning for each major block of questions. Facilitators will use the agenda to ensure that all topic areas and probes are covered in the way we intended them to be covered. Questions may be added or deleted or altered in subsequent interviews as dictated by previously collected data (e.g. data saturation, emergent topics/themes) and in accordance with good qualitative methodology.

*Intent: The intent of these interviews is to examine telehealth practices, facilitators/barriers, lessons learned during implementation, and successful/abandoned strategies used to engage with OAs. We will review the participants’ characteristics prior to the interview: specialty, years in practice, age. We will collect participants’ reflections about their experience with telehealth.*

**Semi-structured interview protocol**

**General:**

*Intent: Prior to enrollment, participants will have provided verbal informed consent allowing us to collect their information on audio. We will explain how long the interview will last and what to expect; therefore, this section of the agenda is created to put the interviewee at ease and gather general impressions of their experiences with telehealth during COVID-19.*

1. Overall, how did you feel about continuing medical care for your patients during COVID-19?
2. Tell me about any experience you had with telehealth before COVID-19.
3. How did your use of remote care practices (e.g. use of *phone calls,* *virtual visits, home monitoring with wearables, app-based management, and web portals)* change after the start of social distancing*?*
4. Describe your experiences with making changes to accommodate new infection control and social isolation mandates.

**Practical considerations (what/where/when/how)**

*Intent: The intent of this section is to understand participants’ practical steps taken to start or continue telehealth and answer basic questions that detail adjustments they had to make for their practice.*

1. In the beginning, how did you feel about your ability to carry out communication, disease monitoring and treatment during COVID-19?
   1. prompt: how did that change as the pandemic continued into summer 2020?
2. Describe the steps you/your practice took to adapt to the virtual/remote setting?
   1. Prompt: have locations of work shifted?
   2. Prompt: have your clinicians’ schedules shifted?
   3. Prompt: what do your clinicians like or dislike about this?
   4. Prompt: Are these changes viable in the long run?
3. When did you adopt telehealth?
   1. If not yet adopted, do you plan to adopt telehealth? When?
4. Help us understand your/your practice’s trajectory of telehealth use.
   1. Please describe why you adopted telehealth.
   2. [if appropriate] Tell me what went into your decision to abandon or stop using telehealth?
   3. [if appropriate] Tell me what went into your decision to continue to use telehealth?
5. Describe for me what mix of telehealth and in person care you provide – 50/50?, 25/75?

**Barriers/Facilitators**

*Intent: The intent of this section is to ask participants to describe how hard/easy it was to use the telehealth equipment or what their expectations about ease of use are and why they had those expectations. Explore how the tech savviness of individuals may have played a role in them effectively pivoting to telehealth.*

1. How hard or easy was it to initiate telehealth using [platform practice chose]?
   1. If not yet adopted, do you expect that initiating telehealth will be hard/easy? (probe for Rationale)
   2. How would you describe your own tech savviness? Did this effect your ability to use telehealth?
2. What concerns do you have about your patients adopting to remote care, if any? (probe for Rationale)
   1. Describe your patient panel. (e.g. primarily Medicare vs. Medicaid vs. private, low-income, affluent, white vs. URM, rural vs. urban)
3. In your experience, what patients struggle most with telehealth? (probe for Rationale)
4. What adverse effects did you experience in initiating telehealth?
5. What challenges did you encounter in continuing care with established patients?
6. What challenges did you encounter in initiating or with new patients?

**Usability**

*Intent: Here, we want participants to describe their level of independence with using telehealth. In general, how reliant were they on others to assist them with use. This section also attempts to ascertain how self-efficacy to use telehealth changed over time and how that may improve usability or not.*

1. How did you feel about your ability to use telehealth when you first started?
   1. Describe what it was like for you to turn it on…get clinical data with it…make a diagnosis…and prescribe treatments…document your note.
2. How do you feel about your ability to use telehealth now that you have used it?
   1. Did anything in particular influence your opinion?
3. How was the quality of support/training you received before telehealth initiation?
4. How was your connectivity to mobile phones/Wi-Fi?
5. What modes did you use? (prompts: *phone calls,* *virtual visits, home monitoring with wearables, app-based management, and web portals) (Probe for Rationale)*
   1. *Prompt: Did the mode vary by “type” of patient?*
6. What tools did you use to improve your assessment, monitoring or treatment? (prompts: *pulse oximeter, home blood pressure monitor, smartwatch, COVID-19 home test kits? Glucometers?*)

**Older Adult Adaptations**

*Intent: In order to better understand how to adapt to challenges or unique situations older adults may face, we will ask about their experience using telehealth with their older patients. In particular, we are interested in teaching methods, type of support, or tricks they employed to aid their use.*

1. What did you do differently when connecting with your older patients?
   1. What problems did you experience?
   2. How do you feel about continuing to use telehealth in this population? What makes you want to continue or stop? (probe for Situational context/Rationale)
2. What, in particular, helps you provide telehealth to older patients?
3. How did you include caregivers in the visit?
4. How did you communicate with hearing impaired/blind/cognitively impaired/non-English speaking?

**Lessons learned**

*Intent: In order to better understand what content we should provide in a telehealth training program for physicians, we will ask questions about lessons learned during implementation.*

1. What advice do you have for other providers/practices as they initiate or maintain telehealth?
2. What challenges still exist/plans to overcome them?

**Appendix 2. Table 2.** Themes and Representative Quotations from Interviews in the Study of Geriatricians, Primary and Emergency Care Physicians on Telehealth, 2020 (Domain: Care Experience)

| **Theme and Subthemes** | **Representative quotations** | |
| --- | --- | --- |
| *Theme 1: Telehealth could transform care delivery, but equitable access must be addressed* | | |
| Telehealth facilitates provider-patient communication | “[S]ince I care for older adults, it's easier for communication and patient care to be done, especially in like assisted living. So, they're able to reach out, schedule an appointment with me and then see the patient at the comfort of their home or assisted living." (Interview 1, PCP*, West, community) | “Don’t you think that there are plenty of elderly people that are very comfortable with this? And definitely there are adult children [who] are comfortable with it. Then the younger generation is going to be comfortable with it. So, I think it’s here to stay.” (Interview 6, geriatrician, West, community) |
| Transforming healthcare | "I can spend more time answering questions and I feel like I can go a little bit even more in depth than I can in emergency department, because I can see multiple people at the same time and talk to five people at the same time." (Interview 23, EM^†^, South, community) | “We can see a large number of patients in a shorter time. There aren't a lot of wait times, and potentially then just volume. I really think is the biggest positive, as well as, patient satisfaction.” (Interview 38, EM, Midwest, academic) |
| Telehealth improves patient access to care | "I think that access that we're giving our patients to care trumps the potential pitfalls that we could otherwise see. […] I know a lot of other offices had to close because of COVID exposures and positive work staff or doctors. We were able to maintain our safety and maintain our numbers, our patients still got care. They still got to be seen. " (Interview 48, PCP, South, community) | "That barrier to reconnecting is so low, and I think that's probably the biggest shift and the biggest help in the telemedicine side of things, is that access. But I mean access in general, people who have a visit, that second, that check in in two days, that check in in a week that you would never do otherwise is really, really nice." (Interview 2, PCP/clinical informatics, West, community) |
| Telehealth is a more patient-centered method of delivering care | “Medicare eliminating the need for them to be face-to-face, actual in-person, and allowing us to do their face to face for transitioning care or certifying their home health through this type of visit as well. It’s like really helpful. It’s less stress for families having to haul either demented or really immobile patients into the office. But still be able to get the care that they need.” (Interview 40, PCP, West, community) | “I don't want to drag a 95-year-old gentleman who's bedbound or basically in his wheelchair all the way to clinic so I can refill his medications or mostly talk to the family member about behavioral disturbances or something that doesn't need a physical, that really doesn't need to be brought in. I think that's [a] disservice to the patient. I think there are opportunities for telemedicine that's wonderful." (Interview 14, geriatrician, West, academic) |
| Patient satisfaction | "[S]ay that the patients that I spoke to are largely happy with the ability to have access to their provider via telehealth. I think that the physicians and [alternative practice providers] APPs^‡^ that I've talked to, or that is communicated this way, have been happy.” (Interview 38, EM, Midwest, academic) | "I think that one concern is that it won't continue to be paid for after [the pandemic], and that many patients will adapt to it. […] So, I hope that, again, that this can continue to be compensated care because that is the expectation of the patients that are pleased with it." (Interview 40, PCP, West, community) |
| Patient-level challenges to accessing telehealth need to be addressed to achieve equity in medicine | "I think this pandemic has shown me how much energy and effort has gone into magnifying those [marginalized] voices in general over the past decades, such that there are really equitable policies in place for healthcare. I think a lot of that has been thrown out the window or undone specifically in this time. Just urging [Congress] to consider how many people lack home internet, lack connected devices, so when they're designing policies about requirements around telehealth or not allowing audio-only diagnoses, just how much - that affects a lot of people." (Interview 34, geriatrician, South, community) | "I think we need like national priority around getting more equitable access to high quality internet. And then I think we need like some sort of core of tech savvy people to like go to people's homes and offer to set up their Wi-Fi for them set up their devices for them and help get them online because it would solve not only the access to care, but the access to senior centers, running zoom Tai-Chi classes and to connecting with family members. So, I think that piece of, of there being the infrastructure to support the connection is really critical. " (Interview 12, geriatrician, Northeast, academic) |
| *Theme 2: Regulatory and policy changes are necessary to improve the older patient experience* | | |
| Privacy | “Because the HIPAA^§^ requirements were lifted, I had to be like, ‘[…] Please don’t call me at other times, but let’s FaceTime on my cell phone so that I can see your face so we can do the visit and keep you safe at home.’” (Interview 20, PCP, West, community) | "Because I think the average person doesn't really care about HIPAA. They don't give a flippa about HIPAA. And so if that being the case, then the opt-out, you have the right to opt-out of non-HIPAA compliant platform." (Interview 3, geriatrician, Northeast, community) |
| Compatibility | “I was able to do an audio video visit today at a nursing home where we had technology issues and compatibility issues. They wanted to use this program, which I couldn’t use, and they don’t want to give me their phone number for me to use the program I want to use.” (Interview 7, geriatrician, South, community) |  |
| Medicolegal protections | “[We] deal with [high]-acuity issues and … incomplete information sometimes and you have to not be set to the same exacting standards or there has to be some protection because [the] patient is accepting some risk by not having physical exam and vitals and full evaluation. I think providers are uncomfortable that they might be solely responsible for missing something that is not potentially a part of the platform and that's why people skewed to very conservative management and low-acuity issues." (Interview 35, EM, Northeast, community) | “What's my level of liability for missing something on an e-visit that maybe I would have caught if I put my hands on the patient." (Interview 33, EM, Midwest, academic) |
| Licensing laws | "If I wanted to work with five different nursing homes, I'd have to be credentialed at five different facilities. And the licensure, if I want to provide care to patients that usually just drive 45 minutes from [adjacent state], but … I technically cannot take care of them." (Interview 46, EM, Northeast, academic) |  |
| Patient-centered policy | "This video or in-person visit is a must for some home health certifications or DME^‖^. […] Those are not very patient centric. For example, if I'm sending a patient to a home health, I know there's a lot of fraud, or over utilization of home health. But I think the therapy team or a home health team are required to do the necessary documentation, sent to primary care. So, I think there's a lot of ways to monitor appropriate use, but making a video or in-person visit… mandatory really just delays care for the patient." (Interview 13, geriatrician, Midwest, academic) | “Just urging [policy makers] to consider how many people lack home internet, lack connected devices, so when they're designing policies about requirements around telehealth or not allowing audio-only diagnoses, just how much - that affects a lot of people." (Interview 34, geriatrician, South, community) |

*PCP, Primary care physician; ^†^EM, Emergency medicine; ^‡^APP, Advanced practice provider; ^§^HIPAA, Health Insurance Portability and Accountability Act; ^‖^DME, Durable medical equipment

**Appendix 3. Table 3.** Themes and Representative Quotations from Interviews in the Study of Geriatricians, Primary and Emergency Care Physicians on Telehealth, 2020 (Domain: Older Individuals’ Health)

| **Themes/Subthemes** | **Representative Quotations** | |
| --- | --- | --- |
| *Theme 3: Telehealth could improve older adults’ health by enhancing access to low-barrier care* | | |
| Telehealth enhances safety related to communicable diseases | “I think originally there was this big worry about healthcare transmission and healthcare workers. Really, this was more about protecting healthcare workers and minimizing transmission of disease […]." (Interview 33, EM*, Midwest, academic) | “I think in flu season, we should keep people home as much as possible. And I don't think this needs to be a coronavirus thing. I wish that now we're well into flu season, I wish people would utilize telehealth visits more.” (Interview 20, PCP^†^, West, community) |
| Telehealth can be an effective early warning system for critical patients | "And when she showed the camera, he was literally laying in bed, not even responsive, she probably would have left him. And I was like, no, you have to call EMS^‡^. And he had had another stroke. … So there's things like that, that you're like, well, I don't know if they would have been caught or not caught based on you being able to …actually see " (Interview 42, PCP, South, community) |  |
| Telehealth can be used to control disease spread in specific settings or address specific conditions | “We've had students who created like a volunteer network to call isolated older adults. And that's felt wonderful to feel like here's a little something for those, you know, 50 older people that we touched to ameliorate that isolation." (Interview 12, geriatrician, Northeast, academic) | "I think especially for people who live in assisted livings... that are worried about the community health in their building, if they leave, they have to be quarantined for 14 days. So, I don't want to contribute to that if I can avoid it, so [telehealth is] very helpful. Yeah, and there are just some patients who were already sort of difficult to transport to begin with, so it's been helpful for that too." (Interview 5, geriatrician, West, community) |

*EM, Emergency medicine; ^†^PCP, Primary care physician; ^‡^EMS, Emergency medical services

**Appendix 4. Table 4.** Themes and Representative Quotations from Interviews in the Study of Geriatricians, Primary and Emergency Care Physicians on Telehealth, 2020 (Domain: Cost)

| **Themes/Subthemes** | **Representative Quotations** | |
| --- | --- | --- |
| *Theme 4: Telehealth has potential to result in cost savings so long as unnecessary referrals and tests are avoided* | | |
| Telehealth could reduce costs related to unnecessary hospital visits | "I think it also helps keep people out of the emergency department or out of urgent care when they would rather not go there […] because it's more convenient even when there's not the threat of infection. So, I think that helps a lot, definitely decreases the strain on other systems because you can deal with those simple things." (Interview 35, EM*, Northeast, community) | "I think that [telehealth] would be really beneficial for patient care and would even be able to prevent a lot of maybe unnecessary ER^†^ visits and hospitalizations. So, I do think that there is a role [for telehealth] in the future. I hope this continues to be something that is approved and funded…after COVID." (Interview 32, geriatrician, Midwest, community) |
| Diagnostic uncertainty related to telehealth could lead to increased ordering of tests and specialty referrals | “If I just saw them in person, I wouldn't necessarily think about ordering all these things, but because I can't actually physically examine them, it makes me more conservative because I feel like, 'Oh, well you should probably go get some kind of imaging.'" (Interview 41, EM, Northeast, academic) | "So I don't know if sometimes in some ways it induces demand for specialty care that I wouldn't normally give because I could handle it. [...] I think there's some concerns that I over-refer because like a derm[atology] consult, which if I saw it in person, I can get a better sense and I would tell them to just watch it versus telling them to follow up with the dermatologist." (Interview 41, EM, Northeast, academic) |
| *Theme 5: Cost-related factors were cited as the driving force in telehealth adoption* | | |
| Sustainability depends on continued reimbursement | "I think it's entirely going to be a funding question because the patients love it and I think providers really like it too." (Interview 4, geriatrician, South, academic) | “... there has to be more reimbursement for performing [telehealth] and even performing it in not necessarily traditional ways like audio only or text-based platforms in some capacity, because that’s what almost exclusively drives whether it’s going to exist or not and without getting reimbursed, it’ll just evaporate again.” (Interview 35, EM, Northeast, community) |
| Lack of payment parity reduced ability to offer telephone visits, which were often necessary to patients for access, and increased tension between administrators and employed physicians | "Our official thing at our organization is that we do not do phone visits, because … phone visits reimburse extremely poorly, generally. So, our official thing is you either come …into the office or do video. […] In reality, we have very limited office visits, and people, the 90-year-olds don't have FaceTime or whatever. The reality is, we're doing a fair number of phone visits [...] And I'm getting yelled at for it, but what am I going to do? So it's been very challenging." (Interview 26, PCP^‡^, Northeast, community) | "Sometimes it's hard to explain why we prioritize the video versus telephonic. Cause even though there's pay parity, it's still not exactly the same.” (Interview 5, geriatrician, West, community) |
| Payment models influenced practice adoption and maintenance of telehealth | "We get paid per member per month. We've never had a disincentive to do video visits or virtual care because we don't get paid per visit for people showing up in our office. […] Actually, we were well on our way to doing video visits and virtual care before COVID ever happened." (Interview 6, geriatrician, West, community) | "Yeah, I think the hospital system is going to get a little nervous because they're not going to be able to do [get] their facility fees. They can't bill for the rent that it takes to keep a clinic open. There has been some pressure … [to] bring some people back in clinic, because we are losing more money. But at what cost and safety? It has to be really thought through." (Interview 14, geriatrician, West, academic) |
| Telehealth payment parity due to COVID-19 influenced physician billing patterns | "If I was entirely RVU-based^§^, I can imagine that I would do what a lot of the folks are doing, which is really try to capture all the revenue that I can.” (Interview 4, geriatrician, South, academic) | "They don't pay as much [for telephone visits], but they have upped reimbursement for it, compared to pre-pandemic times, when you can only get a maximum of I think $12 for a phone call. You had a lot of documentation just to get $12, which was not worth it. We were not trying to bill phone calls back then. And now we are doing some phone visits where we get paid roughly half of what we would otherwise get paid for an audiovisual visit, or an in-person visit, but we still have, patients who are scared to come in and they cannot handle audiovisual technology, so it's just the right thing to do, to have to phone call with them." (Interview 24, geriatrician, South, academic) |
| Telehealth barriers emphasize need for alternative payment models for telehealth | "One other smaller barrier would be these high deductible plans where if you have high copays for each of these things, and you wanted to do these multiple follow ups, yeah, telemedicine breaks the fee for service world. But I think it, again, bumps the value-based care model world." (Interview 2, PCP/clinical informatics, West, community) | "I think [value-based care] would be really great. [We all know] that the hardest patients aren't the simple fracture reductions or the [pause] laceration repairs that actually get compensated, and the really hard thing to do is to have a legitimate relatively lengthy complicated discussion with a family member. And I think that's the part where I really have to use my training and I really have to be careful with how I'm speaking. And I think it has a much bigger effect on patient care, and patient outcomes, and allows me to do the right thing for the patient. And I'm lucky in that I don't work in an RVU-based setting, so I feel like I do what I think needs to be done. […] But for physicians who need to meet certain quotas or where they're really being watched carefully on how much revenue they generate, those things can kind of decrease in priority… I think that's really sad and terrible for our medical system. " (Interview 19, EM, Northeast, community) |
| Needs for payment reform differ by setting and specialty | “Making the laws easier to partner with paramedic services and making sure that paramedic services can be adequately compensated, not just for hospital transfers or 911 calls, but for helping us out and partnering with us on efforts like this. Because if I can send a paramedic to a house, get some vital signs and give them medication, yeah, I can do a whole lot and avoid a whole bunch of unnecessary ER stays. " (Interview 37, EM, Northeast, community) |  |

*EM, Emergency medicine; ^†^ER, Emergency room; ^‡^ PCP, Primary care physician; ^§^RVU, Relative value unit

**Appendix 5. Table 5.** Themes and Representative Quotations from Interviews in the Study of Geriatricians, Primary and Emergency Care Physicians on Telehealth, 2020 (Domain: Workforce Engagement)

| **Themes/Subthemes** | **Representative Quotations** | |
| --- | --- | --- |
| *Theme 6: Telehealth was beneficial for workforce engagement* | | |
| Telehealth satisfaction was in part due to recognition of previously uncompensated care | “I would love it if they were permanent, because I feel like I was already doing all this work, but not getting reimbursed. And we, of course, didn't have the video capability before, so I would just be doing it via messages with patients, which isn't the best way to do it. And then, if I was calling, of course, I was calling on my own time, and I sometimes don't have time for that. So having a dedicated appointment slot where I can address the issue is so much better. I mean, it's better for the patients because they get my undivided attention and time, and lots of times they don't need to come in and they want to be on their couch talking to me. So it's a win-win for both. […] I would recommend that we continue to be able to use telehealth as a major way of treating patients. I mean, it increases patient access to the doctor, definitely. I mean, there's patients I maybe would not have been able to get to if it wasn't through telehealth. And doctors are happier that we can be actually reimbursed for our time.” (Interview 44, PCP*, Midwest, community) | "I mean, we weren't doing telemedicine in the context of video visits [prior to the pandemic]. We were doing telephone care sometimes, we weren't getting paid for it. I think that there's been a shift in our attitudes of like we're doing valuable work and we need to be getting paid for it. Now we have this option that we can get the patient on the schedule instead of calling them at the end of my day, when I'm donating my time for free, I can actually put them on my schedule and get credit for the work that I'm doing here and taking care of them. So I think everybody's attitude and my clinic has been much more favorable toward telemedicine just because we feel like it gives us this opportunity to take care of patients remotely and set a new expectation for them that they're going to get our time, but that there's going to be value assigned to that." (Interview 40, PCP, West, community). |
| Telehealth use led to improved quality of life and work-life balance for physicians | “I hope that it's here to stay. I think that in the future, in my perfect world, I'm going to do two days virtual and three days in person or two days virtual and three and a half days in person or something like that. I think that it's giving, not only for my quality of life, because I am more likely to exercise if I'm working from home. I am more likely to get a healthy meal on the table in a timely manner. My kids are more likely to get picked up from school on time. There's a lot of benefits from just a personal and social aspect that's been helpful for me.” (Participant 32, PCP, South, community) | “I have little kids, one of them doesn't have childcare cause that happens a lot now, you know, then it's easier to be able to do it from home. So that I think has been a positive thing for me to have that flexibility, but also hard in terms of having such a big team and not being co-located with them. So definitely like figuring out the right platform so we can communicate easily with each other has made a big difference.” (Interview 12, geriatrician, Northeast, academic) |
| Telehealth use led to lower work satisfaction due to lack of in-person interactions | “I don't like it [telehealth] too much. I miss seeing my patients in person. […] I also actually miss just the human contact with my staff. I actually think it helps with burnout to see people in person and to be able to walk around and stretch. Even if it's just walking from one room to another room.” (Interview 30, geriatrician, West, academic) |  |

*PCP, Primary care physician
